# Anti-Biofilm Activities from Marine Cold Adapted Bacteria Against Staphylococci and *Pseudomonas aeruginosa*

**DOI:** 10.3389/fmicb.2015.01333

**Published:** 2015-12-14

**Authors:** Rosanna Papa, Laura Selan, Ermenegilda Parrilli, Marco Tilotta, Filomena Sannino, Georges Feller, Maria L. Tutino, Marco Artini

**Affiliations:** ^1^Department of Public Health and Infectious Diseases, Sapienza UniversityRome, Italy; ^2^Department of Chemical Sciences, University of Naples Federico IINaples, Italy; ^3^Laboratory of Biochemistry, Centre for Protein Engineering, University of LiègeLiège, Belgium

**Keywords:** Polar bacteria, anti-virulence, anti-biofilm molecules, anti-adhesive, non-biocidal agents

## Abstract

Microbial biofilms have great negative impacts on the world’s economy and pose serious problems to industry, public health and medicine. The interest in the development of new approaches for the prevention and treatment of bacterial adhesion and biofilm formation has increased. Since, bacterial pathogens living in biofilm induce persistent chronic infections due to the resistance to antibiotics and host immune system. A viable approach should target adhesive properties without affecting bacterial vitality in order to avoid the appearance of resistant mutants. Many bacteria secrete anti-biofilm molecules that function in regulating biofilm architecture or mediating the release of cells from it during the dispersal stage of biofilm life cycle. Cold-adapted marine bacteria represent an untapped reservoir of biodiversity able to synthesize a broad range of bioactive compounds, including anti-biofilm molecules. The anti-biofilm activity of cell-free supernatants derived from sessile and planktonic cultures of cold-adapted bacteria belonging to *Pseudoalteromonas, Psychrobacter*, and *Psychromonas* species were tested against *Staphylococcus aureus, Staphylococcus epidermidis*, and *Pseudomonas aeruginosa* strains. Reported results demonstrate that we have selected supernatants, from cold-adapted marine bacteria, containing non-biocidal agents able to destabilize biofilm matrix of all tested pathogens without killing cells. A preliminary physico-chemical characterization of supernatants was also performed, and these analyses highlighted the presence of molecules of different nature that act by inhibiting biofilm formation. Some of them are also able to impair the initial attachment of the bacterial cells to the surface, thus likely containing molecules acting as anti-biofilm surfactant molecules. The described ability of cold-adapted bacteria to produce effective anti-biofilm molecules paves the way to further characterization of the most promising molecules and to test their use in combination with conventional antibiotics.

## Introduction

The great ability of bacteria to colonize new environments can be linked, in most cases, to their capacity to develop a protective architecture called biofilm. The biofilm lifestyle is associated with a high tolerance to exogenous stress, and treatment of biofilms with antibiotics or other biocides is usually ineffective at eradicating them (Hall-Stoodley and Stoodley, 2009). Biofilm formation is therefore a major problem in many fields, ranging from the food industry to medicine (López et al., 2010; Høiby et al., 2011). It is worth mentioning that, in medical settings, biofilms are the cause of persistent infections implicated in 80% or more of all microbial cases-releasing harmful toxins and even obstructing indwelling catheters (Epstein et al., 2012).

Staphylococci are recognized as the most frequent causes of biofilm-associated infections (Otto, 2008). *Staphylococcus aureus* (*S. aureus*) is an opportunistic dangerous pathogen that can cause serious diseases in humans, ranging from skin and soft tissue infections to invasive infections of the bloodstream, heart, lungs and other organs. A statistical study showed that 30% of U.S. population was colonized by *S. aureus* (Nicholson et al., 2013). In addition, 1.5% of U.S. population was found to be a carrier of methicillin-resistant *S. aureus* (MRSA) that is a major cause of healthcare-related infections, responsible for significant proportion of nosocomial infections worldwide. Recently in the U.S., deaths from MRSA infections have surpassed those from many other infectious diseases, including HIV/AIDS (Nicholson et al., 2013).

*Staphylococcus epidermidis*, conventionally considered as a commensal bacterium of human skin, it can cause significant problems when breaching the epithelial barrier, especially during biofilm-associated infection of indwelling medical devices (Dohar et al., 2009; Rogers et al., 2009). Most diseases caused by *S. epidermidis* are of a chronic character and occur as device-related infections (such as intravascular catheter or prosthetic joint infections) and/or their complications (Rogers et al., 2009).

*Pseudomonas aeruginosa* (*P. aeruginosa*) is an important pathogen responsible for infections in patients who suffer from respiratory diseases (Saxena et al., 2014) like cystic fibrosis (CF). Recurrent and chronic respiratory tract infections in CF patients result in progressive lung damage and represent the primary cause of morbidity and mortality. *P. aeruginosa* can cause hard to treat life threatening infections due to its high resistance to antibiotics and to the ability to form antibiotic tolerant biofilms.

The development of anti-biofilm strategies is therefore of major interest and currently constitutes an important field of investigation in which non–biocidal molecules are highly valuable to avoid the rapid appearance of escape mutants.

From another point of view, the biofilm could be considered as a source of novel drugs and holds great potential due to the specific physical and chemical conditions of its ecosystem. For example, the production of extracellular molecules that degrade adhesive components in the biofilm matrix is a basic mechanism used in the biological competition between phylogenetically different bacteria (Brook, 1999; Wang et al., 2007, 2010). These compounds often exhibit broad-spectrum biofilm-inhibiting or biofilm-detaching activity when tested *in vitro* and their use in a combination therapy with antibiotics could be of interest.

Marine bacteria are a resource of biologically active products (Debbab et al., 2010). Cold-adapted marine bacteria represent an untapped reservoir of biodiversity endowed with an interesting chemical repertoire. A preliminary characterization of molecules isolated from cold-adapted bacteria revealed that these compounds display antimicrobial, anti-fouling and various pharmaceutically relevant activities (Bowman, 2007). The ability of Polar marine bacteria, belonging to different genera/species, to synthesize bioactive molecules might represent the results of the selective pressure to which these bacteria are subjected. One of the developed survival strategies may be represented by the production of metabolites with anti-biofilm activity, which might be exploited to fight the biological competition of other bacteria.

Recently, we observed that Antarctic marine bacterium *Pseudoalteromonas haloplanktis* TAC125 produces and secretes several compounds of biotechnological interest (Papaleo et al., 2013), including molecules inhibiting the biofilm of the human pathogen *S. epidermidis* (Papa et al., 2013b; Parrilli et al., 2015). This activity impairs biofilm development and disaggregates the mature biofilm without affecting bacterial viability, showing that its action is specifically directed against biofilm (Papa et al., 2013b; Parrilli et al., 2015).

In this work we evaluated the anti-biofilm activity of supernatants derived from cultures of cold-adapted bacteria belonging to *Pseudoalteromonas, Psychrobacter*, and *Psychromonas* genera. Supernatants were obtained from bacterial cultures made both in sessile and planktonic conditions. The potential anti-biofilm activity was tested on bacterial cultures of *P. aeruginosa* PAO1, three different strains of *S. aureus* and three different strains belonging *S. epidermidis* species. The results obtained highlighted that several supernatants show anti-biofilm activity against most species analyzed. Preliminary evaluations on the physico-chemical nature of the molecules responsible for anti-biofilm activity emphasized their different nature.

## Materials and Methods

### Bacterial Strains and Culture Conditions

Bacterial strains used in this work are listed in **Table 1**. Bacteria were grown in Brain Heart Infusion broth (BHI, Oxoid, UK). Biofilm formation was assessed in static conditions. Planktonic cultures were grown in flasks under vigorous agitation (180 rpm). Cold-adapted bacteria were grown at 15°C, while staphylococci and *P. aeruginosa* were grown at 37°C.

**Table 1:** Strains used in this study.

Strain	Origin	Reference and/or source
*Pseudoalteromonas haloplanktis* TAA207	Antarctic sea water*^a^* (marine sediment)	Liège collection
*Pseudoalteromonas haloplanktis* TAE56	Antarctic sea water*^a^* (algae necrosed suspended in sea water)	Liège collection
*Pseudoalteromonas haloplanktis* TAE57	Antarctic sea water^a^ (algae necrosed suspended in sea water)	Liège collection
*Pseudoalteromonas haloplanktis* TAE79	Antarctic sea water*^a^* (algae necrosed suspended in sea water)	Liège collection
*Pseudoalteromonas haloplanktis* TAE80	Antarctic sea water*^a^* (algae necrosed suspended in sea water)	Liège collection
*Psychrobacter sp.*TAD1	Antarctic sea water*^a^*	Liège collection
*Psychrobacter sp.*TAD18	Antarctic sea water*^a^* (frozen algae)	Liège collection
*Pseudoalteromonas haloplanktis* TAB87	Antarctic sea water*^a^*	Liège collection
*Psychrobacter arcticus* 273-4	Siberian permafrost sediment cores	Bakermans et al., 2006
*Psychromonas arctica*	Arctic seawater (Svalbard islands, Arctic)	Groudieva et al., 2003
*Staphylococcus aureus* 6538P	Clinical isolate	ATCC collection
*Staphylococcus aureus* 25923	Clinical isolate	ATCC collection
*Staphylococcus aureus* 20372	Clinical isolate from septic arthritis	ATCC collection
*Staphylococcus epidermidis* RP62A	Reference strain isolated from infected catheter	ATCC collection
*Staphylococcus epidermidis* O-47	Clinical isolate from septic arthritis	Heilmann et al., 1996
*Staphylococcus epidermidis* XX-17	Clinical isolate from infected catheter	Our collection
*Pseudomonas aeruginosa* PAO1	Clinical isolate from wound	ATCC collection

*^a^Isolated from Antarctic coastal sea water sample collected in the vicinity of the French Antarctic station Dumont d’Urville, Terre Adélie (66°40′ S; 140° 01′ E).*

### Biofilm Formation of Polar Bacteria

Biofilm formation of cold-adapted bacteria was obtained at 15°C in BHI (Oxoid, UK). The wells of a sterile 24-well flat-bottomed polystyrene plate were filled with 1 ml of BHI, and an opportune dilution of bacterial culture in exponential growth phase (about 0.1 OD 600 nm) was added into each well. The plates were aerobically incubated up to 96 h at 15°C in static condition, measuring biofilm formation each 24 h. After the removal of spent medium and of not adhered cells and rinsing with PBS, adhered cells were stained with 0.1% crystal violet, rinsed twice with double-distilled water, and thoroughly dried as previously described (Papa et al., 2013a). The dye bound to adherent cells was solubilized with 20% (v/v) glacial acetic acid and 80% (v/v) ethanol. The absorbance of each well was measured at 590 nm. Each data point is composed of four independent experiments performed in triplicate.

### Preparation of Cell-free Supernatants from Cold-adapted Bacteria

The cell-free supernatants of a liquid culture of cold-adapted strains grown in sessile condition were designated as SNB, while the cell-free supernatants of a liquid culture of psychrophilic strains grown in planktonic condition were designated as SNP.

For the preparation of SNB, wells of a sterile 24-well flat-bottomed polystyrene plate were filled with 900 μl of BHI and 100 μl of each overnight bacterial culture was added into each well. The plates were incubated at 15°C monitoring biofilm formation each 24 h. After 96 h, supernatants were recovered and centrifuged at 13000 rpm at 4°C for 30 min. Supernatants were sterilized by filtration through membranes with a pore diameter of 0.22 μm, and stored at 4°C until use.

For the preparation of SNP bacterial cultures were grown in planktonic conditions at 15°C under vigorous agitation (180 rpm) for 24 h. Supernatants were recovered by centrifugation at 13000 rpm at 4°C and processed as described above.

### Biofilm Formation of Staphylococci and *Pseudomonas*

Biofilm formation of *Staphylococcus* and *Pseudomonas* species was evaluated in the presence of SNB and SNP supernatants, respectively. Quantification of *in vitro* biofilm production was based on method previously reported (Artini et al., 2015). Briefly, the wells of a sterile 96-well flat-bottomed polystyrene plate were filled with 100 μl of the appropriate medium. 1/100 dilution of overnight bacterial cultures was added into each well (about 5.0 OD 600 nm). Each well was filled with 50 μl of BHI and 50 μl of each supernatant, respectively. In this way each supernatant was used diluted 1:2 with a final concentration of 50%. As control, the first row contained bacteria grown only in 100 μl of BHI (untreated bacteria). The plates were incubated aerobically for 24 h at 37°C.

Biofilm formation was measured using crystal violet staining. After treatment, planktonic cells were gently removed; each well was washed three times with PBS and patted dry with a piece of paper towel in an inverted position. To quantify biofilm formation, each well was stained with 0.1% crystal violet and incubated for 15 min at room temperature, rinsed twice with double-distilled water, and thoroughly dried. The dye bound to adherent cells was solubilized with 20% (v/v) glacial acetic acid and 80% (v/v) ethanol. After 30 min of incubation at room temperature, OD_590_ was measured to quantify the total biomass of biofilm formed in each well. Each data point is composed of three independent experiments each performed at least in eight-replicates.

### Surface Coating Assay

A volume of 25 μl of cell-free supernatant (SNB or SNP), or 25 μl of saline as control, was deposited to the center of a well of a 24-well tissue-culture-treated polystyrene microtiter plate. The plate was incubated at 37°C for 1 h to allow complete evaporation of the liquid. The wells were then filled with 1 ml of broth containing 10^4^–10^5^ CFU/ml of *S. epidermidis* O-47 and incubated at 37°C. After 18 h, wells were rinsed with water and stained with 1 ml of 0.1% crystal violet. Stained biofilms were rinsed with water and dried, and the wells were photographed.

### Physico-chemical Characterization of Anti-biofilm Compounds

The heat sensitivity of anti-biofilm compounds were evaluated by incubating the culture supernatants (SNB or SNP), for 1 h in a water bath at 50°C and cooled on ice. For the protease treatment, proteinase K (Sigma Aldrich, St Louis, MO, USA) was added to aliquots of supernatants at a final concentration of 1 mg/ml and the reactions were incubated for 1 h at 37°C. As controls, supernatants were incubated for 1 h at 37°C without proteinase K, a treatment which did not impair the anti-biofilm activities. For each of the above tests, the anti-biofilm activities of treated and untreated culture supernatants were compared using the microtiter plate assay against staphylococci and *P. aeruginosa* PAO1, respectively. Each data point is composed of three independent experiments performed in six-replicates.

### Statistics and Reproducibility of Results

Data reported were statistically validated using Student’s *t*-test comparing mean absorbance of treated and untreated samples. The significance of differences between mean absorbance values was calculated using a two-tailed Student’s *t*-test. A *p*-value of <0.05 was considered significant.

## Results

### Cold-adapted Bacteria Biofilm Formation

Biofilm formation of Polar bacteria was evaluated at 15°C in BHI at different times as described in material and methods section. Bacteria were grown in static condition in the same medium used for staphylococci and *P. aeruginosa* cultures to avoid interference in the following experiments due to the medium composition. The biofilm-forming ability of Polar bacterial strains was tested by a quantitative assay. The best production of biofilm was obtained by incubating the cells in static condition for 96 h at 15°C (data not shown). Almost all studied bacteria are able to form biofilm with different capabilities (**Table 2**). For example, in the tested condition, *Pseudoalteromonas haloplanktis* TAE80 and *Psychrobacter arcticus* 273-4 seemed to be unable to produce biofilm, while *Psychromonas arctica* was found to be a strong biofilm producer, as already reported (Vishnivetskaya et al., 2000; Groudieva et al., 2003).

**Table 2:** Biofilm formation of the investigated bacterial strains.

Strain	Biofilm (OD 590 nm)
TAA207	0.41 ± 0.09
*Pseudoalteromonas haloplanktis* TAE56	0.15 ± 0.06
*Pseudoalteromonas haloplanktis* TAE57	0.20 ± 0.10
*Pseudoalteromonas haloplanktis* TAE79	0.90 ± 0.20
*Pseudoalteromonas haloplanktis* TAE80	0.03 ± 0.02
*Psychrobacter sp.*TAD1	0.60 ± 0.20
*Psychrobacter sp.*TAD18	0.90 ± 0.20
*Pseudoalteromonas haloplanktis* TAB87	0.90 ± 0.20
*Psychrobacter arcticus* 273-4	0.09 ± 0.07
*Psychromonas arctica*	11.00 ± 1.00
*Staphylococcus aureus* 6538P	1.10 ± 0.10
*Staphylococcus aureus* 25923	1.90 ± 0.30
*Staphylococcus aureus* 20372	0.80 ± 0.20
*Staphylococcus epidermidis* RP62A	1.10 ± 0.10
*Staphylococcus epidermidis* O-47	2.10 ± 0.20
*Staphylococcus epidermidis* XX-17	0.69 ± 0.06
*Psychrobacter aeruginosa* PAO1	2.40 ± 0.50

*Each data point is composed of four independent experiments performed in triplicate. Standard errors are reported.*

### Effect of Exoproducts Derived from Cold-adapted Cultures on Biofilm Formation of Different Pathogen

The anti-biofilm effects of cold-adapted bacterial culture supernatants grown at 15°C either in planktonic or sessile conditions were examined on different pathogens: *P. aeruginosa* PAO1, three strains belonging to *S. epidermidis* species, and three strains belonging to *S. aureus* species (**Table 1**).

The specific environmental conditions prevailing within biofilms induce profound genetic and metabolic rewiring of the biofilm-dwelling bacteria and can allow the production of metabolites different from those obtained in planktonic condition. Therefore, supernatants deriving from sessile growths were designated as B letter, while supernatants deriving from planktonic cultures under vigorous agitation were designated as P letter, respectively.

In order to exclude that the tested Polar supernatants contain molecules affecting bacterial viability, the 20 cell-free supernatants were analyzed also for antimicrobial activity. An opportune dilution (10^6^cfu/ml were used as reported by National Committee for Clinical Laboratory Standards NCCLS, 2004) of each bacterial culture of *S. aureus* and *P. aeruginosa* in exponential phase was seeded on TSA plates. Each plate was spotted with Polar cell free supernatant separately and incubated at 37°C for 20 h. No antimicrobial activity on *S. aureus* and *P. aeruginosa* strains was highlighted for all tested supernatants (data not shown).

Anti-biofilm effect is reported as percentage of residual biofilm after treatment in comparison with untreated bacteria. In some cases an increase of biofilm formation was highlighted after the treatment.

**FIGURE 1:**
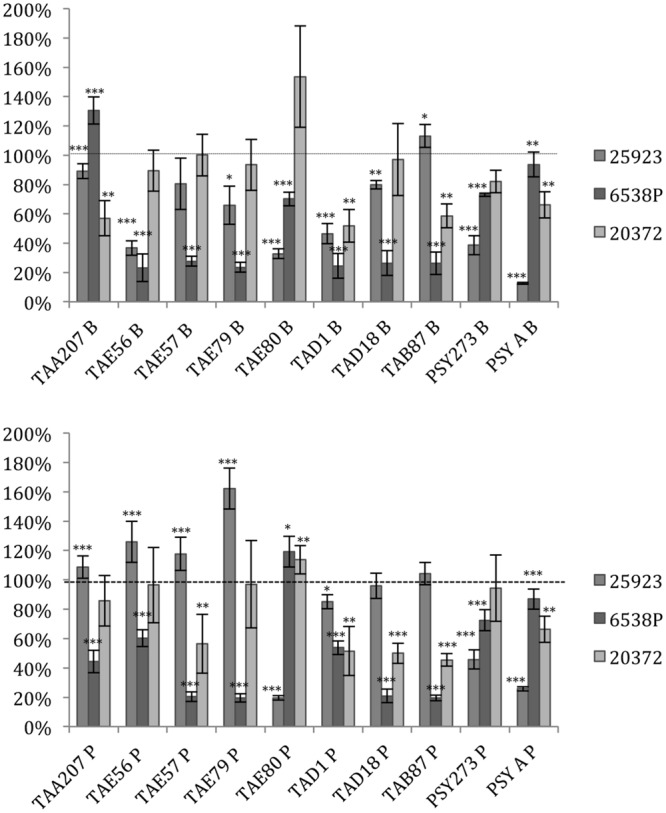
**Effect of Polar supernatant treatment on biofilm formation for three strains of *Staphylococcus aureus*.** Data are reported as percentage of residual biofilm after the treatment. Biofilm formation was considered unaffected in the range 90–100%. Differences in mean absorbance were compared to the untreated control and considered statistically significant when *p* < 0.05 (^∗^*p* < 0.05, ^∗∗^*p* < 0.01, ^∗∗∗^*p* < 0.001) according to Student’s *t*-test.

Several supernatants of Polar bacteria have anti-biofilm activity against all *S. aureus* tested strains (**Figure 1**, Supplementary Table S1). *S. aureus* 6538P showed a reduction in biofilm formation when treated with cold-adapted bacteria supernatants except in the case of TAA207 B and TAE80 P supernatants. TAE80 and PSYA supernatants deriving from both sessile and planktonic growth conditions showed a good anti-biofilm effect on *S. aureus* 25923, a reference strain for CF infections (Alhanout et al., 2011) (**Figure 1**). Three supernatants (TAD1 B, TAD18 P, TAB87 P) allowed a reduction of *S. aureus* 20372 biofilm higher than 50% (**Figure 1**). As shown in **Figure 1**, supernatants derived from sessile and planktonic cultures showed differences in their ability to prevent *S. aureus* biofilm formation and the effect of each supernatant is strictly strain-specific. Indeed, in such cases, the same supernatant is able to impair biofilm formation of one strain rather than others belonging to the same bacterial species; for example, TAD18 P is able to inhibit the biofilm formation of *S. aureus* 6538P and *S. aureus* 20372 but it is of not effective on *S. aureus* 25923. It is interesting to note that supernatants derived from sessile and planktonic cultures of the same Polar bacterium showed differences in their ability to prevent *S. aureus* biofilm formation, for example TAE80 B supernatant is able to impair biofilm of *S. aureus* 6538P while TAE80 P induces a significant increase in the biofilm formation. In addition TAE79 B, but not TAE79 P, produces an anti-biofilm molecule able to inhibit the biofilm formation of *S. aureus* 25923. On the contrary TAE79 P treatment increases the biofilm production of 25923 strain. It is worth mentioning that one supernatant, i.e., TAD1 B, is quite effective in interfering with biofilm formation of all tested *S. aureus* strains.

**FIGURE 2:**
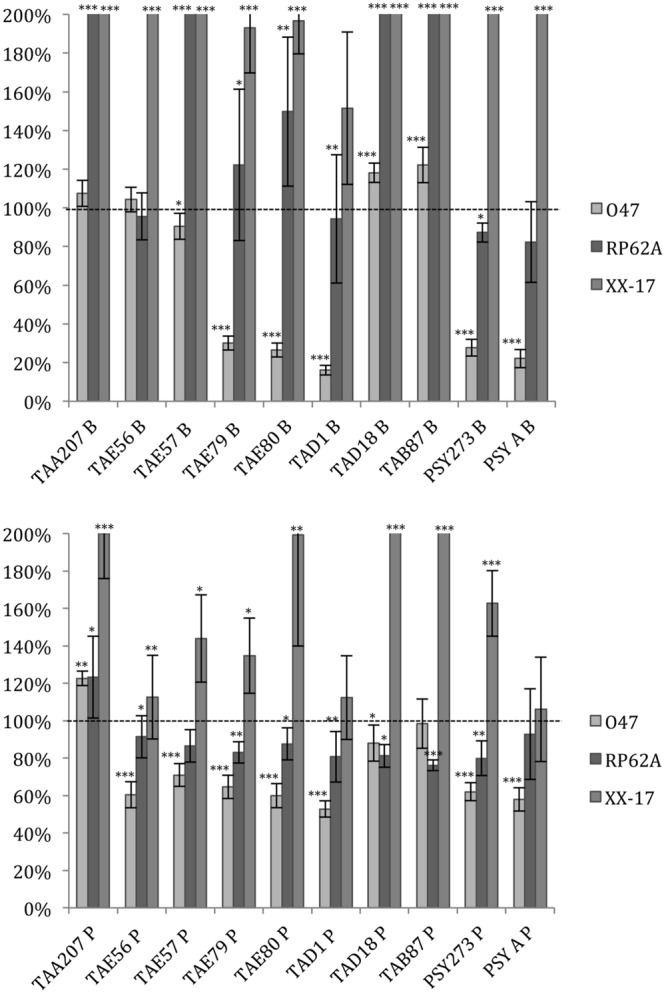
**Effect of Polar supernatant treatment on biofilm formation for three strains of *S. epidermidis*.** Data are reported as percentage of residual biofilm after the treatment. Biofilm formation was considered unaffected in the range 90–100%. Differences in mean absorbance were compared to the untreated control and considered statistically significant when *p* < 0.05 (^∗^*p* < 0.05, ^∗∗^*p* < 0.01, ^∗∗∗^*p* < 0.001) according to Student’s *t*-test.

As far as *S. epidermidis* is concerned (**Figure 2**), in most cases the treatments induced an increase in biofilm formation, except for *S. epidermidis* O-47 strain treated with TAE79, TAE80, TAD1, PSY273 and PSYA supernatants derived from planktonic and sessile cultures where a strong reduction was evidenced (**Figure 2**).

Also in the case of *S. epidermidis*, supernatants derived from sessile and planktonic cultures of the same Polar bacterium showed differences in their ability to prevent biofilm formation. Cell free supernatant of TAE56P is able to inhibit the biofilm of *S. epidermidis* O-47 while TAE56B has no effect, indicating that the anti-biofilm molecule is produced only when the cells are grown in planktonic condition.

**FIGURE 3:**
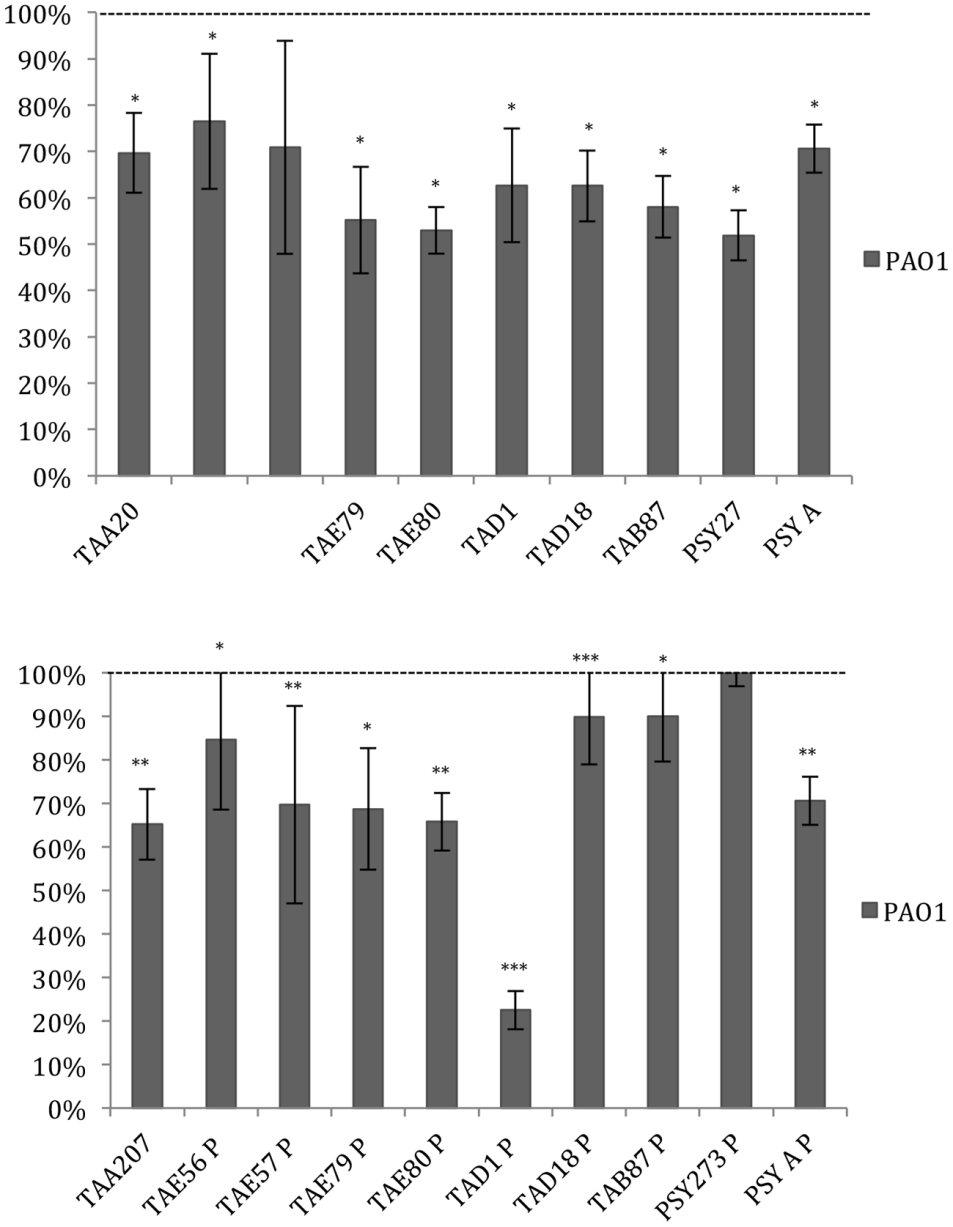
**Effect of Polar supernatant treatment on biofilm formation for *P. aeruginosa* PAO1.** Data are reported as percentage of residual biofilm after the treatment. Biofilm formation was considered unaffected in the range 90–100%. Differences in mean absorbance were compared to the untreated control and considered statistically significant when *p* < 0.05 (^∗^*p* < 0.05, ^∗∗^*p* < 0.01, ^∗∗∗^*p* < 0.001) according to Student’s *t*-test.

Data reported in **Figure 3** demonstrated that the ability of *P. aeruginosa* PAO1 to form biofilm is affected by all cold-adapted supernatants deriving from sessile cultures with a rate of reduction between 30 and 50%. Only TAD1 P is able to reduce the *P. aeruginosa* PAO1 biofilm more than 70%. An additional control experiment was performed for *P. aeruginosa* in order to exclude a dilution effect on the bacterial growth after the supplementation with each supernatant due to diverse nutrient concentration between the untreated bacteria and the treated ones. In particular, as growth medium was also used BHI 2X concentrated. This experiment was performed in addition to the standard condition because we noted an inhibitory effect on biofilm formation when *P. aeruginosa* was treated with all supernatants. Data obtained with BHI twofold concentrated were nearly superimposable excluding an effect due to the diverse nutrient concentration between the treated and untreated bacteria (data not shown).

### Physico-chemical Characterization of Anti-biofilm Compounds from Polar Bacteria

To determine a preliminary chemical characterization of biofilm-inhibiting compounds, cell free supernatants of cold-adapted bacteria were dispensed in several aliquots, and submitted to chemical (proteinase K) and physical (thermal) treatments. Percentage of biofilm inhibition of each treated aliquot was determined on *S. epidermidis* O-47, *S. aureus* 6538P and *P. aeruginosa* PAO1 biofilms (**Table 3**). Data are reported as the percentage of anti-biofilm activity remaining after each treatment compared to the effect of the same untreated supernatant. As shown in **Table 3**, the proteinase K treatment reduced the anti-biofilm activity of tested supernatants on *S. epidermidis* O-47 and *S. aureus* 6538P biofilms, while this treatment did not interfere with their anti-biofilm ability on *P. aeruginosa* PAO1 except for TAD18 B, indeed the proteinase K treatment reduced its anti-biofilm activity at value less than 10%.

**Table 3:** Effect of physico-chemical treatments on the anti-biofilm activity of cold-adapted bacteria supernatants on *S. epidermidis* O-47, *S. aureus* 6538P and *P. aeruginosa* PAO1, respectively.

	Proteinase K treatment			Heat treatment		
	*S. epidermidis*	*S. aureus*	*P. aeruginosa*	*S. epidermidis*	*S. aureus*	*P. aeruginosa*
TAA207 P	ND	<10%	100%	ND	<10%	100%
TAE56 P	<10%	<10%	ND	90%^NS^	<10%	ND
TAE57 P	<10%	<10%	80%^∗∗^	100%	<10%	100%
TAE79 P	<10%	<10%	85%^∗∗^	100%	<10%	100%
TAE80 P	<10%	ND	70%^∗∗∗^	100%	ND	100%
TAD1 P	<10%	<10%	85%^∗∗^	80%	<10%	100%
TAD18 P	ND	<10%	ND	ND	<10%	ND
TAB87 P	ND	<10%	ND	ND	<10%	ND
PSY273 P	<10%	<10%	ND	90%^NS^	<10%	ND
PSYA P	<10%	ND	100%	90%^NS^	ND	100%
TAA207 B	ND	ND	100%	ND	ND	90%^NS^
TAE56 B	ND	<10%	100%	ND	40%	100%
TAE57 B	ND	<10%	70%	ND	<10%	100%
TAE79 B	<10%	<10%	80%	80%^∗^	<10%	100%
TAE80 B	<10%	<10%	100%	90%^NS^	<10%	100%
TAD1 B	<10%	<10%	100%	60%^∗∗^	<10%	100%
TAD18 B	ND	<10%	<10%	ND	50%	100%
TAB87 B	ND	<10%	80%^NS^	ND	<10%	100%
PSY273 B	<10%	<10%	70%^∗∗^	100%	<10%	100%
PSYA B	<10%	ND	100%	100%	ND	100%

*With P letter was designated the cell-free supernatants of cold-adapted strains grown in planktonic condition; with B letter was designated the cell-free supernatants of cold-adapted strains grown in sessile condition. Data are reported as percentage of residual activity compared with each untreated supernatant. Differences in mean absorbance were compared to the untreated control and considerend significant when *p* < 0.05 (^∗^*p* < 0.05, ^∗∗^*p* < 0.01, ^∗∗∗^*p* < 0.001; NS, Not Significant) according to Student’s t-test. ND, not determined.*

Furthermore, thermal treatment at 50°C significantly reduced the anti-biofilm effect of almost all supernatants on *S. aureus* but did not impair their activity on *P. aeruginosa* and *S. epidermidis*. This latter suggests that each supernatant contains different molecules with anti-biofilm activity that works selectively and independently on different bacterial species.

### Anti-biofilm Surfactant Activity of Polar Compounds

To assess the ability of cell free Polar bacteria supernatants to modify the surface properties of an abiotic substrate, a surface coating assay was performed. Evaporation coating was used to deposit each supernatant onto the surface of polystyrene wells, and then the ability of the coated surfaces to repel biofilm formation by *S. epidermidis* O-47 was tested. This latter pathogen was selected for this assay as it is the strongest biofilm producer amongst the bacteria used in this work and because it is able to preferentially form biofilm on the surface while *P. aeruginosa* typically forms biofilm at the liquid/air interface.

**FIGURE 4:**
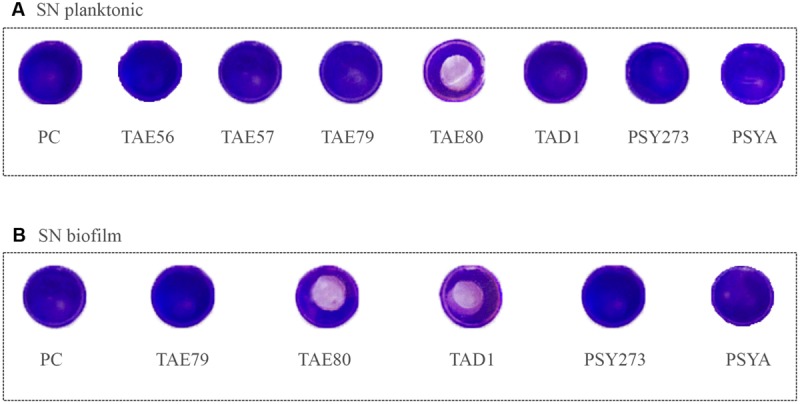
**Analysis of surfactant capability of each Polar supernatant on *S. epidermidis* O-47.** The center of each well of a 24-well tissue-culture-treated polystyrene microtiter plate was coated with each supernatant. After evaporation, the wells were then filled with staphylococci and incubated at 37°C. Then wells were rinsed with water and stained with 1 ml of 0.1% crystal violet. Stained biofilms were rinsed with water and dried, and the wells were photographed. **(A)** Supernatants derived from planktonic growths. **(B)** Supernatants derived from biofilm growths.

As clearly visible in **Figure 4**, TAE80 supernatants derived from both planktonic and sessile cultures and TAD1 supernatant derived from only sessile growth, were able to repel biofilm formation specifically only in the area where the supernatants were deposited, indicating that they contain molecules acting as anti-biofilm surfactants.

## Discussion

In this paper the attention was focused on anti-biofilm molecules produced by cold-adapted marine bacteria since they represent an untapped reservoir of biodiversity and a potential source of molecules able to inhibit pathogens biofilm formation. The target pathogens chosen were *P. aeruginosa, S. aureus*, and *S. epidermidis.*

Biofilm is a key element in *S. epidermidis, S. aureus*, and *P. aeruginosa* infectious processes, but the matrix composition and molecules involved in attachment, development and detachment phases in these three bacterial species, are very different (Joo and Otto, 2012). Further, pathways and regulation of quorum sensing systems in these three strains are deeply different (Solano et al., 2014).

In staphylococci biofilm formation depends on a complex interplay of several elements such as adhesins, extracellular matrix binding proteins, biofilm associated proteins, proteins involved in PIA synthesis (*icaADBC*), autolysins (Alt), etc. *Staphylococcus* strains used in this work were chosen on the basis of different characteristics. In particular, *S. aureus* ATCC 6538P is a reference strain for antimicrobial testing; *S. aureus* ATCC 25923 and ATCC 20372 are clinical isolates. As for their ability to form biofilm, *S. aureus* strains were classified as reported: ATCC 25923 is a strong biofilm producer, ATCC 6538P is a medium/strong biofilm producer and ATCC 20372 is a medium/weak biofilm producer according to Cafiso et al. (2007).

Several cold adapted bacteria produce molecules able to interfere with *S. aureus* biofilm formation. These molecules display a different efficiency on different *S. aureus* tested strains and in all strains the anti-biofilm molecules seems to be proteinaceous. On the contrary only few Polar strains produce anti-biofilm molecules active on *S. epidermidis* O-47 and RP62A biofilms, and none are able to interfere with *S. epidermidis* XX-17 biofilm formation. It is important to underline that XX-17 strain produces a biofilm characterized by a polysaccharide *ica*-independent poorly characterized so far.

*Staphylococcus epidermidis* RP62A is a reference strain isolated from infected catheter; *S. epidermidis* XX-17 and O-47 are clinical isolates. The clinical isolate O-47 is a naturally occurring non-functional *agr* mutant characterized by a frameshift mutation within *agrC* (Vuong et al., 2003) while the *S. epidermidis* XX-17 is an *ica* defective mutant (Artini et al., 2013). Determination of *S. epidermidis* biofilm formation showed a strong production for the O-47 strain, medium/strong production for the reference strain RP62A and a medium/weak biofilm formation for the XX-17 strain defined according to the literature (Cafiso et al., 2004). Moreover, the six staphylococcal strains considered here were previously investigated to assess the presence of genes coding for various proteins involved in adhesion and biofilm formation (Artini et al., 2013).

In *P. aeruginosa* the biofilm matrix is totally different, because the bacterium produces three exopolysaccharides, the glucose-rich Pel polysaccharide (Friedman and Kolter, 2004), the mannose-rich Psl polysaccharide (Friedman and Kolter, 2004), and alginate (Govan and Deretic, 1996). In particular, for *P. aeruginosa* we used the reference strain PAO1 since the biofilm characterization of this strain was previously reported (Yang et al., 2005).

The reported differences in biofilm features of the three pathogens could explain the different ability of cold adapted bacteria supernatants to impair their biofilm formation. It is interesting to note that, in all reported cases, the supernatants proved to be non-biocidal and specifically directed against biofilm.

All studied Polar strains are able to produce anti-biofilm molecules against *P. aeruginosa* biofilm. Furthermore in all cases the anti-biofilm molecules seems to have the same chemical-physical features (were not heat-labile and seem to have a non-protein nature), except in case of TAD18 B. These results could suggest that the molecule responsible for the anti-biofilm activity is the same for all cold-adapted strains. In particular, Polar anti-biofilm molecules involved in the inhibition of *P. aeruginosa* biofilm could be polysaccharides or a small molecule acting as quorum sensing inhibitors. Several studies have identified different bacterial polysaccharides and signaling molecules that inhibit biofilm formation by wide spectrum of bacteria including *P. aeruginosa* (Valle et al., 2006; Wittschier et al., 2007; Kim et al., 2009).

The increase of biofilm production following the treatment with such supernatants is an interesting result. This latter strengthens the hypothesis regarding the production of bacterial molecules able to regulate the biofilm formation inter- and intra- species in different environmental niches. The regulatory pathways of this phenotype could be linked to competition dynamics of extreme habitats (i.e., Polar niches). The identification of the molecules responsible for these mechanisms could be interesting and also open new perspectives for the control of bacterial biofilm formation. It is worth to note that “row” supernatants that we used represent a complex pool of chemical cues that could be characterized by different capabilities either responsible for impair biofilm formation and increase it.

Data reported in this paper demonstrate that anti-biofilm activity of cold-adapted bacteria supernatants deriving from planktonic and sessile cell cultures display several differences in terms of specificity and efficiency. Some biofilm-specific metabolites previously reported (Gjersing et al., 2007; Booth et al., 2011; Yeom et al., 2013) may exhibit an antagonist effect against competing microorganisms. Indeed, several studies showed that bacterial biofilm constitute untapped sources of natural bioactive molecules antagonizing adhesion or biofilm formation of other bacteria (Papa et al., 2013b; Rendueles et al., 2013). Furthermore, differences between activity of supernatants, derived from sessile and planktonic cultures, could be linked to a different concentration of active molecules produced in these two growth conditions. This latter could be particularly relevant if the active molecules are involved in quorum sensing signaling.

Moreover, in this paper we report that several cold-adapted strains (TAD1, TAE79, TAE80, PSY273 and PSYA), belonging to different genera, are able to produce different anti-biofilm molecules active against *S. epidermidis, S. aureus* and *P. aeruginosa* biofilms.

The preliminary chemical characterization of the anti-biofilm molecules indicates that the same bacterium produces different molecules active against different targets. For example, TAD1 produces a thermo-stable protein active against *S. epidermidis* biofilm, a thermo-labile protein active against *S. aureus* biofilm, and a non-proteinaceus molecule able to impair *P. aeruginosa* biofilm. Furthermore the supernatants of TAD1 deriving from biofilm and planktonic growth showed also a different behavior in surface coating assay, suggesting the production of an anti-biofilm surfactant molecule only when TAD1 is grown in sessile form. Also for the supernatants deriving from TAE80 growths were evidenced the presence of different anti-biofilm molecules that are able to specifically act against the different bacterial species tested. In particular, we analyzed the dose dependent profile of TAE80 supernatants deriving form planktonic and biofilm growths tested against strongest biofilm producers belonging the three different species (**Supplementary Figure S1**). Both TAE80 supernatants (TAE80B and TAE80P) showed an anti-biofilm activity clearly dose-dependent against *S. aureus* 25923 and *S. epidermidis* O-47 while their activity against *P. aeruginosa* does not seem to be dose-dependent.

The ability of cold-adapted marine bacteria to produce several anti-biofilm molecules could suggest that the capacity to avoid the biofilm and colonization of competitor bacteria is a selective advantage in this extreme environment. Besides their ecological meaning, the anti-biofilm molecules from cold-adapted bacteria may have interesting biomedical applications combined with conventional antibiotics in order to eradicate biofilm infection.

## Conflict of Interest Statement

The authors declare that the research was conducted in the absence of any commercial or financial relationships that could be construed as a potential conflict of interest.

## Supplementary Material

Figure S1 **A dose-dependent effect on biofilm formation of *S. aureus* 25923 **(A)**, *S. epidermidis* O-47 **(B)**, *P. aeruginosa* PaO1 **(C)** in the presence of scalar concentration of TAE80 B and TAE80 P, respectively (starting from 50%).** Results are representative of three independent experiments. UN, untreated samples.
