# Supplementary material for: Anti-Biofilm Activities from Marine Cold Adapted Bacteria Against Staphylococci and Pseudomonas aeruginosa
Source: Front Microbiol. 2015 Dec 14;6:1333. doi: 10.3389/fmicb.2015.01333 (PMC4677098; doi:10.3389/fmicb.2015.01333)
Supplement: Supplementary file 1 [file Table_1.DOCX]

**Table 1S**

Effect of Polar supernatant treatment on biofilm formation for three strains of *S. aureus*, *S. epidermidis* and one strain of *P. aeruginosa*. Data are reported as percentage of residual biofilm after the treatment. Each data point is composed of three independent experiments each performed at least in 8-replicates and is reported as mean and standard deviation.

|  | **TAA207B** | **TAE56B** | **TAE57B** | **TAE79B** | **TAE80B** | **TAD1B** | **TAD18B** | **TAB87B** | **PSY273B** | **PSYAB** |
| --- | --- | --- | --- | --- | --- | --- | --- | --- | --- | --- |
| ***S. aureus* 6538P** | 130,50 ± 9,20 | 23,22 ± 8,96 | 27,71 ± 3,35 | 23,50 ± 3,25 | 70,15 ± 5,36 | 24,50 ± 9,10 | 26,42 ± 8,11 | 26,23 ± 7,80 | 73,01 ± 1,18 | 93,64 ± 8,15 |
| ***S. aureus* 20372** | 56,96 ± 11,92 | 89,53 ± 13,88 | 100,17 ± 13,89 | 93,44 ± 17,12 | 153,58 ± 35,16 | 51,82 ± 10,93 | 97,02 ± 25,24 | 58,60 ± 8,24 | 82,03 ± 8,34 | 66,10 ± 9,02 |
| ***S. aureus* 25923** | 89,17 ± 5,06 | 36,73 ± 5,32 | 80,48 ± 17,00 | 65,94 ± 13,48 | 32,80 ± 2,98 | 46,48 ± 6,99 | 79,90 ± 2,98 | 113,15 ± 8,48 | 38,62 ± 6,21 | 12,65 ± 1,01 |
| ***S. epidermidis* RP62A** | 498,84 ± 42,96 | 95,56 ± 12,40 | 268,02 ± 27,31 | 122,26 ± 38,73 | 149,73 ± 38,20 | 94,22 ± 32,84 | 499,35 ± 83,78 | 342,16 ± 23,78 | 87,21 ± 5,22 | 82,37 ± 21,21 |
| ***S. epidermidis* O-47** | 107,54 ± 6,81 | 104,35 ± 6,02 | 90,41 ± 7,34 | 30,15 ± 4,37 | 26,49 ± 4,01 | 16,15 ± 3,06 | 118,19 ± 4,89 | 122,22 ± 8,89 | 27,64 ± 4,01 | 22,11 ± 5,12 |
| ***S. epidermidis* XX-17** | 417,61 ± 22,16 | 471,00 ± 15,15 | 379,21 ± 59,82 | 193,13 ± 22,89 | 196,76 ± 17,23 | 151,55 ± 39,21 | 533,76 ± 24,91 | 568,72 ± 28,89 | 208,99 ± 4,38 | 210,38 ± 5,00 |
| ***P. aeruginos*a PAO1** | 69,68 ± 8,67 | 76,50 ± 14,98 | 70,88 ± 22,90 | 55,17 ± 12,25 | 52,94 ± 5,32 | 62,68 ± 12,34 | 62,54 ± 7,69 | 58,03 ± 7,12 | 51,89 ± 4,86 | 70,60 ± 5,18 |
|  | **TAA207P** | **TAE56P** | **TAE57P** | **TAE79P** | **TAE80P** | **TAD1P** | **TAD18P** | **TAB87P** | **PSY273P** | **PSYAP** |
| ***S. aureus* 6538P** | 44,27 ± 7,60 | 60,23 ± 6,18 | 20,34 ± 2,99 | 19,56 ± 2,98 | 119,26 ± 11,29 | 53,81 ± 5,06 | 20,86 ± 5,11 | 19,43 ± 2,11 | 72,48 ± 7,08 | 86,88 ± 6,78 |
| ***S. aureus* 20372** | 85,66 ± 17,21 | 96,46 ± 26,04 | 56,45 ± 20,17 | 96,99 ± 30,04 | 113,74 ± 9,97 | 51,51 ± 17,11 | 49,96 ± 7,00 | 45,45 ± 4,34 | 94,31 ± 23,22 | 66,22 ± 9,01 |
| ***S. aureus* 25923** | 108,66 ± 7,55 | 125,91 ± 13,88 | 117,71 ± 11,28 | 162,27 ± 13,89 | 19,60 ± 2,27 | 85,14 ± 5,08 | 95,97 ± 9,11 | 104,26 ± 8,18 | 45,75 ± 6,73 | 25,76 ± 1,11 |
| ***S. epidermidis* RP62A** | 123,18 ± 21,85 | 91,42 ± 10,98 | 86,55 ± 8,99 | 83,12 ± 5,97 | 87,60 ± 8,88 | 80,68 ± 12,99 | 81,25 ± 6,12 | 76,20 ± 3,06 | 79,93 ± 8,89 | 92,83 ± 24,34 |
| ***S. epidermidis* O-47** | 122,62 ± 3,88 | 60,31 ± 7,25 | 70,95 ± 6,31 | 64,59 ± 5,99 | 59,92 ± 6,16 | 52,80 ± 3,84 | 88,09 ± 10,34 | 98,46 ± 13,18 | 62,04 ± 4,89 | 57,90 ± 6,16 |
| ***S. epidermidis* XX-17** | 200,08 ± 24,12 | 112,60 ± 22,01 | 143,94 ± 22,67 | 134,70 ± 20,08 | 199,37 ± 58,97 | 112,29 ± 21,97 | 262,32 ± 32,77 | 309,05 ± 31,98 | 162,66 ± 18,11 | 106,10 ± 28,32 |
| ***P. aeruginos*a PAO1** | 65,18 ± 8,14 | 84,58 ± 15,79 | 69,68 ± 23,18 | 68,71 ± 13,86 | 65,77 ± 6,82 | 22,51 ± 3,79 | 89,91 ± 10,09 | 90,03 ± 10,11 | 108,23 ± 11,05 | 70,60 ± 5,45 |
